# Reemergence of Chikungunya Virus in Bo, Sierra Leone

**DOI:** 10.3201/eid1907.121563

**Published:** 2013-07

**Authors:** Rashid Ansumana, Kathryn H. Jacobsen, Tomasz A. Leski, Andrea L. Covington, Umaru Bangura, Mary H. Hodges, Baochuan Lin, Alfred S. Bockarie, Joseph M. Lamin, Moses J. Bockarie, David A. Stenger

**Affiliations:** Njala University, Bo, Sierra Leone (R. Ansumana, A.S. Bockarie);; Mercy Hospital Bo (R. Ansumana, A.L. Covington, U. Bangura, A.S. Bockarie, J.M. Lamin);; Liverpool School of Tropical Medicine, Liverpool, United Kingdom (R. Ansumana, M.H. Hodges, M.J. Bockarie);; George Mason University, Fairfax, Virginia, USA (K.H. Jacobsen);; US Naval Research Laboratory, Washington, DC, USA (T.A. Leski, B. Lin, D.A. Stenger)

**Keywords:** chikungunya virus, CHIKV, viruses, reemerging infectious disease, Sierra Leone, Africa

## Abstract

We diagnosed 400 possible IgM-positive cases of chikungunya virus in Bo, Sierra Leone, during July 2012–January 2013 by using lateral flow immunoassays. Cases detected likely represent only a small fraction of total cases. Further laboratory testing is required to confirm this outbreak and characterize the virus.

Outbreaks of infection with chikungunya virus (CHIKV), an alphavirus that is transmitted by bites of infected *Aedes* spp. mosquitoes, were frequent in sub-Saharan Africa and southern and Southeast Asia during the 1950s–1970s, but the infection largely disappeared in the 1980s; only sporadic cases were observed (*1*). The virus reemerged in the early 2000s; major outbreaks were reported in Kenya, some island nations in the Indian Ocean, and several countries in Asia (*2*,*3*).

The primary symptoms of CHIKV infection are high fever (>38.5°C [102°F]) and severe pain in the distal joints of the extremities or the lumbar spine. A maculopapular rash, sensorineural impairment, severe headache, and other nonspecific symptoms may also occur. Symptoms usually resolve within 1–2 weeks after onset of fever, but for a sizeable proportion of patients, arthralgia and arthritis become chronic and pain persists for years (*2*,*3*).

A nationwide serosurvey in Sierra Leone in 1972 detected cases of CHIKV infection throughout the country (*4*), but we are not aware of any cases reported since the mid-1970s. Two recent developments made reemergence appear imminent. First, outbreaks of reemerging CHIKV have been reported in neighboring Guinea (*5*) and in Senegal (*6*). Second, recent yellow fever cases in Sierra Leone have shown that *Aedes* spp. mosquito–borne infections are common (*7*). Thus, it was not surprising when we initiated an infectious disease surveillance study in July 2012 in the city of Bo, in Southern Province, Sierra Leone, that we detected possible chikungunya virus infections. We report initial results of our investigation.

## The Study

On July 7, 2012, the Mercy Hospital Research Laboratory (MHRL) in Bo, Sierra Leone, initiated a 1-year infectious disease surveillance program to identify the diversity of pathogens causing febrile illness in the city. A tiered analysis approach was used. First, all specimens from febrile study participants were tested for ≈12 infections with various pathogens, including CHIKV, by commercially available test kits. Specimens that showed negative results in this first round of testing were further tested by using cultures, multiplex PCR, and resequencing pathogen microarrays. The research protocol was approved by Njala University, George Mason University, the Liverpool School of Tropical Medicine, the US Naval Research Laboratory, and the Sierra Leone Ethics and Scientific Review Committee.

During July 7, 2012–January 10, 2013, MHRL conducted first-tier lateral flow immunoassay (LFI) tests of blood samples from all 932 outpatients ≥5 years of age who had been clinically examined at the hospital, were found to have febrile illness, and consented to having blood drawn for laboratory testing. LFI test kits (SD Bioline; Standard Diagnostics, Inc., Seoul, South Korea) were used for diagnosis of IgM against CHIKV; IgG and IgM against dengue virus and hepatitis A virus; hepatitis B virus surface antigen, hepatitis C virus, HIV-1/2, and antibodies against these viruses; and IgG and IgM against *Leptospira* spp., *Salmonella enterica* Serovar typhi, and syphilis.

Most patients reported that they had sought medical care within several days after the onset of their febrile illnesses. Levels of IgM against CHIKV are usually detectable by immunochromatographic methods within a few days after infection and persist for ≈3–4 months (*1*,*2*). The LFI test kits for CHIKV were reported by the manufacturer to have a sensitivity of 97.1% and a specificity of 91.1% compared with those of ELISA (*8*). An independent evaluation found a sensitivity of 50.8% and a specificity of 89.2% for the kits; sensitivity ranged from 40.9% 1–5 days after onset of illness to 65.4% 16–20 days after onset (*9*). Specificity decreases after the first week (*10*).

**Figure 1:**
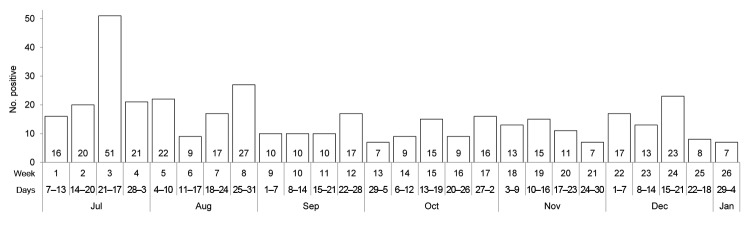
Weekly number of IgM-positive chikungunya virus test results at Mercy Hospital Research Laboratory, Bo, Sierra Leone, July 7, 2012–January 4, 2013.

More than half of the cases tested during the first week of the surveillance program were positive by LFI for CHIKV. Thus, we notified the Sierra Leone Ministry of Health and Sanitation of a possible CHIKV outbreak. By January 10, 2013, 400 (42.9%) of 932 febrile patients were positive by LFI for CHIKV (Figure 1). Ages of the 400 CHIKV IgM-positive patients ranged from 6 years to 85 years; 172 (43.0%) were male patients. Of these 400 patients, 220 (55.0%) reported arthralgia, 189 (47.3%) chills, and 156 (39.0%) headaches. Co-infections were common; 92 (23.0%.) were co-infected with malaria, 37 (9.3%) with HIV, 33 (8.3%) with hepatitis B virus, and smaller numbers with hepatitis A, hepatitis C, tuberculosis, typhoid, and syphilis. Four CHIKV-positive samples were also positive for dengue.

**Figure 2:**
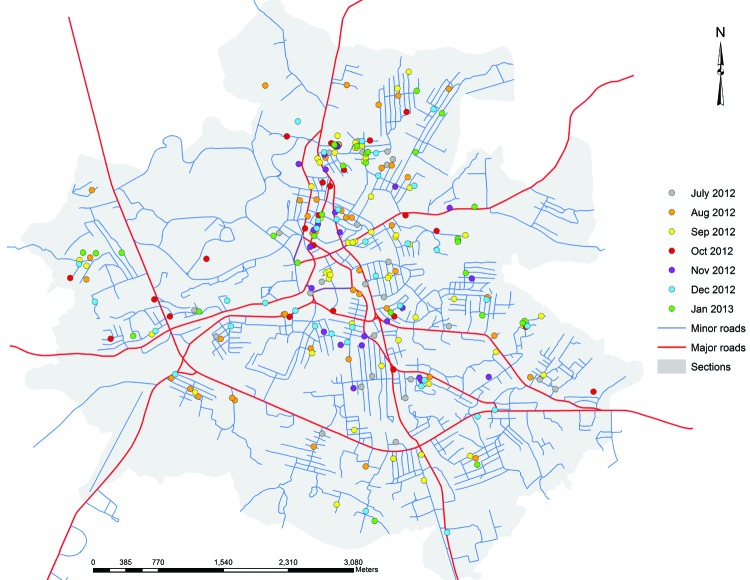
Residence locations for IgM-positive cases of infection with chikungunya virus, Bo, Sierra Leone, July 7, 2012–January 4, 2013.

On July 28, MHRL launched an Ushahidi-based website (www.ushahidi.com) to compile case reports. Details about the patients who were positive for CHIKV were uploaded to the MHRL website (www.mhrlsl.com/GIA/ushahidi) and, if possible, were geolocated on an open street map (www.openstreetmap.org) that linked to a map of Bo created by MHRL for health research purposes (*11*). The map showed that the cases were located throughout Bo (Figure 2). Of the 400 LFI-positive case-patients, 319 (79.8%) could be mapped; the remainder did not provide a home street address on the laboratory patient information form. However, the sample was not population-based because Mercy Hospital is 1 of several hospitals serving Bo, so a city-wide attack rate could not be determined.

Results of attempts by the US Naval Research Laboratory to confirm the LFI results by using semi-nested reverse transcription PCR on fast technique for analysis of nucleic acid–preserved samples were inconclusive, possibly because of genetic sequence variation from well-characterized strains or because of the timing of specimen collections. Viral loads for humans with CHIKV infection decrease after the second day of symptoms, and viral titers may be low after the fifth day (*12**,**13*). Because CHIKV nucleic acids are only detectable in serum for a few days, reverse transcription PCR results are often discordant with those of serologic (IgM and IgG) assays. Confirmation that an outbreak occurred is dependent on isolation of the virus, followed by molecular characterization, full-genome sequencing, and phylogenetic mapping.

## Conclusions

Our results suggest that an outbreak of chikungunya virus occurred in Sierra Leone. The exact time of the reemergence of this virus cannot be pinpointed, but retrospective analyses of outpatient charts suggested that, on the basis of syndromic criteria, the first cases occurred in January 2012 and the outbreak peaked during the rainy season in 2012. Other outbreaks reported in central and west Africa have also occurred during the rainy season, which is typical for *Aedes* spp. mosquito–borne infections (*6*,*14**,**15*). Because Mercy Hospital serves only a relatively small proportion of the residents of Bo, the cases detected likely represent only a small fraction of the total cases that have occurred. Further study will be required to confirm the laboratory results and, if further investigation is warranted, to document the extent of the outbreak.

## References

[1] Ng LC, Hapuarachchi HC. Tracing the path of chikungunya virus: evolution and adaptation. Infect Genet Evol. 2010;10:876–85. 10.1016/j.meegid.2010.07.012 20654736

[2] Burt FJ, Rolph MS, Rulli NE, Mahalingam S, Heise RT. Chikungunya: a re-emerging virus. Lancet. 2012;379:662–71. 10.1016/S0140-6736(11)60281-X 22100854

[3] Staples JE, Breiman RF, Powers AM. Chikungunya fever: an epidemiological review of a re-emerging infectious disease. Clin Infect Dis. 2009;49:942–8. 10.1086/605496 19663604

[4] Mouchet J, Robin Y. Serological and entomological survey on yellow fever in Sierra Leone: report on a mission May 16–June 7, 1972. Report no. AFR/YF/18. Geneva: World Health Organization; 1973.

[5] Jentes ES, Robinson J, Johnson BW, Conde I, Sakouvougui Y, Iverson J, Acute arbovirual infections in Guinea, west Africa, 2006. Am J Trop Med Hyg. 2010;83:388–94. 10.4269/ajtmh.2010.09-0688 20682888 PMC2911191

[6] Thonnon J, Spiegel A, Diallo M, Diallo A, Fontenille D. Epidémies à virus chikungunya en 1996 et 1997 au Sénégal. Bull Soc Pathol Exot. 1999;92:79–82 . 10399593

[7] World Health Organization. Outbreak news: yellow fever, Sierra Leone. Wkly Epidemiol Rec. 2011;86:101–2 . 21442796

[8] Standard Diagnostics, Inc. Chikungunya IgM [cited 2012 Sep 12]. http://www.standardia.com/html_e/mn03/mn03_01_00.asp?intId=119

[9] Kosasih H, Widjaja S, Surya E, Hadiwijaya SH, Butarbutar DP, Jaya UA, Evaluation of two IgM rapid immunochromatographic tests during circulation of Asian lineage chikungunya virus. Southeast Asian J Trop Med Public Health. 2012;43:55–61 . 23082554

[10] Rianthavorn P, Wuttirattanakowit N, Prianantathavorn K, Limpaphayom N, Theamboonlers A, Poovorawan Y. Evaluation of a rapid assay for detection of IgM antibodies to chikungunya. Southeast Asian J Trop Med Public Health. 2010;41:92–6 . 20578487

[11] Ansumana R, Malanoski AP, Bockarie AS, Sundufu AJ, Jimmy DH, Bangura U, Enabling methods for community health mapping in developing countries. Int J Health Geogr. 2010;9:56. 10.1186/1476-072X-9-56 21034454 PMC2987786

[12] Laurent P, Le Roux K, Grivard P, Bertil G, Naze F, Picard M, Development of a sensitive real-time reverse transcriptase PCR assay with an internal control to detect and quantify chikungunya virus. Clin Chem. 2007;53:1408–14. 10.1373/clinchem.2007.086595 17586592

[13] Ray P, Ratagiri VH, Kabra SK, Lodha R, Sharma S, Sharma BS, Chikungunya infection in India: results of a prospective hospital based multi-centric study. PLoS ONE. 2012;7:e30025. 10.1371/journal.pone.0030025 22363413 PMC3281818

[14] Caron M, Paupy C, Grard G, Becquart P, Mombo I, Nso BB, Recent introduction and rapid dissemination of chikungunya virus and dengue virus serotype 2 associated with human and mosquito co-infections in Gabon, Central Africa. Clin Infect Dis. 2012;55:e45–53. 10.1093/cid/cis530 22670036

[15] Demanou M, Antonio-Nkondjio C, Ngapana E, Rousset D, Paupy C, Manuguerra JC, Chikungunya outbreak in a rural area of western Cameroon in 2006: a retrospective serological and entomological survey. BMC Res Notes. 2010;3:128. 10.1186/1756-0500-3-128 20444282 PMC2883987

